# Digital Gangrene: An Unusual Manifestation of Non-Hodgkin Lymphoma

**DOI:** 10.1155/2022/8963753

**Published:** 2022-03-02

**Authors:** Muhammad Shoaib Momen Majumder, Shamim Ahmed, Tajkia Haque, Syed Atiqul Haq, Saumitra Chakravarty, Md. Abu Shahin, Din-E-Mujahid Mohammad Faruque Osmany, Johannes J. Rasker

**Affiliations:** ^1^Department of Rheumatology, Bangabandhu Sheikh Mujib Medical University (BSMMU), Dhaka, Bangladesh; ^2^Department of Pathology, Bangabandhu Sheikh Mujib Medical University (BSMMU), Dhaka, Bangladesh; ^3^Department of Cardiology, Bangabandhu Sheikh Mujib Medical University (BSMMU), Dhaka, Bangladesh; ^4^University of Twente, Faculty of Behavioral, Management and Social Sciences, Department Psychology, Health and Technology, Enschede, Netherlands

## Abstract

**Background:**

Non-Hodgkin lymphomas (NHLs) comprise a group of haematologic malignancies with different histologic subtypes. The clinical picture varies from indolent to aggressive presentation and nodal (lymphadenopathy) to extranodal (central nervous system, gastrointestinal, cutaneous plaque, or ulcer) involvement. Digital gangrene is seldom reported. Here, we describe a patient with pain and blackening of all fingers and toes as presenting symptoms of NHL. *Case Presentation*. A 32-year-old male weaver had been smoking three to five cannabis-containing cigarettes daily for about ten years and methamphetamine four to five tablets daily for five years. He had no history of Raynaud's phenomenon, fever, cough, weight loss, skin rash, joint pain, and atherogenic or thrombogenic risk factors. We found normal blood pressure and absent peripheral pulses in arms and legs, dry gangrene of all fingers and toes, generalized lymphadenopathy, and hepatomegaly with ascites. The chest X-ray was normal, as were blood sugar, lipid profile, and hepatic and renal function. Rheumatoid factor, antinuclear and antiphospholipid antibodies, C-ANCA and P-ANCA, hepatitis B and C, and HIV were negative. CT abdomen revealed hepatosplenomegaly with multiple intra-abdominal lymphadenopathies. The peripheral angiogram showed 90-99% stenosis of radial and dorsalis pedis arteries with normal proximal vessels. Diagnosis of non-Hodgkin lymphoma was confirmed by histopathology of cervical lymph node (diffuse type), immunohistochemically subtyped as peripheral T cell lymphoma (not otherwise specified). The digital ischemia worsened despite cessation of cannabis and methamphetamine and starting CHOP (cyclophosphamide, doxorubicin, vincristine, and prednisolone) treatment, making amputation necessary.

**Conclusion:**

We present, to our knowledge, the first report of peripheral T cell lymphoma, NOS presenting with gangrene in all digits complicated by methamphetamine and cannabis abuse. This uncommon vascular manifestation of non-Hodgkin lymphoma may cause a diagnostic dilemma and delayed initiation of treatment.

## 1. Introduction

Digital ischemia encompasses a broad differential diagnosis. This is commonly associated with systemic autoimmune diseases (systemic sclerosis, systemic lupus erythematosus, Sjogren's syndrome, mixed connective tissue disease, etc.), primary vasculitis (polyarteritis nodosa, cryoglobulinemic vasculitis, granulomatosis with polyangiitis, Takayasu disease, etc.), atherosclerosis, antiphospholipid syndrome, blood dyscrasias (polycythemia rubra vera, paraproteinemia, etc.), and some drugs like vinblastine and bleomycin [1]. Less commonly, the underlying cause may be a malignancy. Possibly, the earliest description of a paraneoplastic syndrome involving the vascular system was mentioned by Trousseau in 1865 [2]. O'Connor first reported the coexistence of digital ischemia and carcinoma in 1884 [3].

Musculoskeletal and rheumatic syndromes can herald an underlying malignancy. Paraneoplastic syndromes are caused by remote effects of the tumour, which are unrelated to tumour mass or distant metastasis. There is a wide array of paraneoplastic syndromes presenting with rheumatic manifestations. Paraneoplastic vascular acrosyndromes (Raynaud's syndrome, acrocyanosis, digital ischemia, and acronecrosis) are less frequently associated with haematological malignancies [4]. Paraneoplastic vasculitis and vascular syndromes have been described in different haematological malignancies like Hodgkin's disease, multiple myeloma, IgA myeloma, myelofibrosis, and hairy cell leukaemia and also in combination with some solid tumours like squamous cell carcinoma of the lung, renal, prostate, and colon carcinoma [5]. The association with non-Hodgkin's lymphomas (NHLs) is very uncommon, and an association of paraneoplastic cutaneous vasculitis and NHL was first reported in 1965 [6]. The pathogenesis of paraneoplastic acral vascular syndromes is probably multifactorial and is poorly understood [4]. Here, we describe a patient presenting with acute digital ischemia in the hands and feet with concomitant substance abuse, eventually diagnosed as non-Hodgkin's lymphoma, immunophenotypically subtyped as peripheral T cell lymphoma, not otherwise specified (PTCL, NOS).

## 2. Case Presentation

A normotensive, nondiabetic 32-year-old male weaver was admitted to our rheumatology department on the 3^rd^ March 2020 with pain and blackening of the fingers and painful, discoloured toes. He had severe excruciating pain in his toes that suddenly started six weeks before admission. He had no cardiovascular risk factors, no intermittent claudication symptoms, no history of Raynaud's phenomenon, and no medical treatment. Initially, his symptoms improved after taking aspirin, clopidogrel, and cilostazol, but the symptoms reappeared on 16^th^ February 2020, initially in his fingers, then after 10 days in his toes. His past medical history was unremarkable, with no history of fever, cough, shortness of breath, weight loss, skin rash, joint pain, or weakness of any part of his body. He gave a history of multiple nodular swellings over his neck, axilla, and groin for the last six months. They were insidious in onset, gradually progressive in size with no pain or other systemic features, but occasionally regressed in size upon intake of homoeopathic treatment.

The patient gave a history of 10 pack-year smoking. He had regularly smoked eight to ten joints (dry marijuana leaves rolled into cigarettes) per day for 10 years. He also had abused yaba (methamphetamine) 4-5 tablets per day for five years. He quitted these (cannabis, methamphetamine, and smoking) habits about 40 days before admission at the very onset of the digital infarction, but the tissue necrosis progressed. He had no family history of cardiovascular diseases. He had sleep disturbance for the last one and a half years.

**Figure 1:**
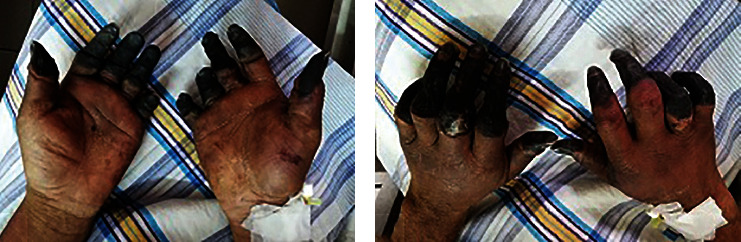
(a, b) Digital gangrene of the fingers.

**Figure 2:**
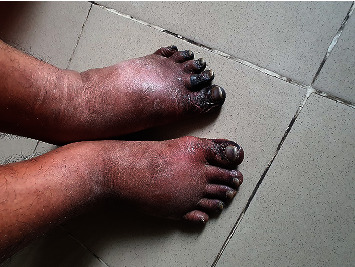
Digital necrosis affecting the toes.

On physical examination, the patient looked toxic with an average physique. There was peripheral oedema. He had a stable respiratory rate of 16 beats per minute, blood pressure of 125/80 mm of Hg in the right and 120/80 mm of Hg in the left arm, and axillary temperature of 98°F (36.67°C). The peripheral pulses were not felt in either upper or lower limbs. The tips of his fingers and toes were cool bilaterally and tendered on palpation. He had dry gangrene in all his fingers, some up to and some beyond the proximal interphalangeal (PIP) joints, and in the distal parts of his toes with clearly demarcated margins (Figures 1(a), 1(b), and 2). His hands were shiny, red, and swollen. The gangrenous involvement of the fingers was more severe than in his toes. He had generalized lymphadenopathy, including the cervical, axillary, and inguinal areas, which were discrete, firm in consistency, nontender, and not fixed to the overlying skin or underlying structures with no discharging sinus. The largest lymph gland was 2 × 3 cm^2^, situated in the axillary area. On abdominal examination, he had hepatomegaly with ascites; the spleen was not palpable. The nervous system examination, including cranial nerves, was normal. A systemic survey of other organs detected no abnormality.

Laboratory investigations showed leucocytosis of 16,000 per microliter (normal value 4,000 to 11,000) with neutrophils 78% (normal value 40-75%) and lymphocytes 17% (normal value 20-50%) with normal haemoglobin level and platelet count. The erythrocyte sedimentation rate (ESR) was 15 mm in the first hour (reference range 0-10) and C-reactive protein of 5.5 mg/L (reference range < 6). A peripheral blood film displayed mature leucocytes with an increased total count and increased neutrophil distribution containing toxic granules within some of them. His blood sugar (6.1 mmol/L), lipid profile (total cholesterol 126, HDL 33, LDL 79, and triglyceride 72 mg/dL), and liver and renal function were within normal limits. The urine routine and microscopy tests were normal. Serum calcium, albumin, and uric acid were within normal limits. Serum electrolyte report revealed sodium 135 mmol/L (reference range 135-148 mmol/L), potassium 3.4 mmol/L (reference range 3.5-5.2 mmol/L), and chloride 98 mmol/L (reference range 95-107 mmol/L). The antiphospholipid and anticardiolipin antibodies, rheumatoid factor (RF), antinuclear antibodies (ANA), and anti-neutrophil cytoplasmic antibodies (C-ANCA and P-ANCA) were all negative. Viral screening for hepatitis B and C and human immune deficiency virus (HIV) was found to be negative. Urine drug screening for amphetamine and cannabis was negative. His serum complement (C_3_) level was normal, and the C_4_ level was just below normal. The electrocardiogram (ECG) displayed a normal rate and rhythm, and the heart was normal at echocardiography. The whole abdomen's ultrasonogram showed hepatosplenomegaly, ascites, a space-occupying lesion in the right lobe of the liver, possibly a haemangioma, and multiple abdominal lymphadenopathies. The chest X-ray and ECG showed no abnormality.

**Figure 3:**
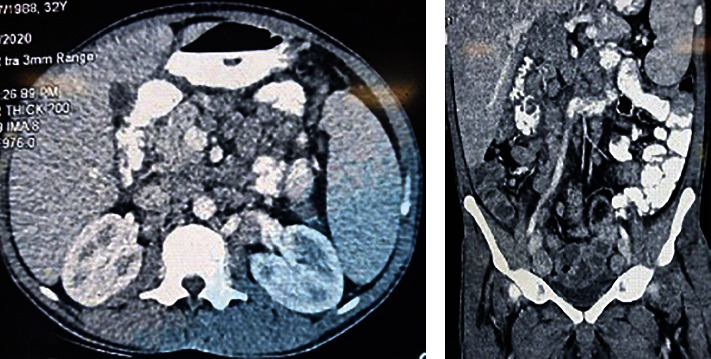
(a) Axial view of CT scan of the abdomen showing hepatosplenomegaly with pre- and para-aortic mesenteric lymphadenopathy; (b) enlarged liver and spleen with multiple intra-abdominal lymphadenopathies.

**Figure 4:**
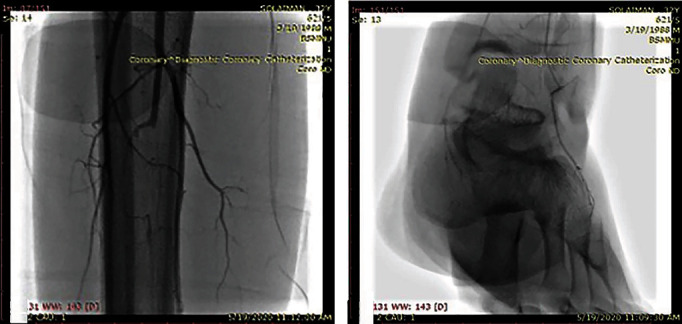
(a) Arteriography showing nonvisualized right anterior tibial artery from the middle part of the tibia. Note the absence of corkscrew collaterals or microaneurysm; (b) the arteria dorsalis pedis and the dorsal arch are nonvisualized.

**Figure 5:**
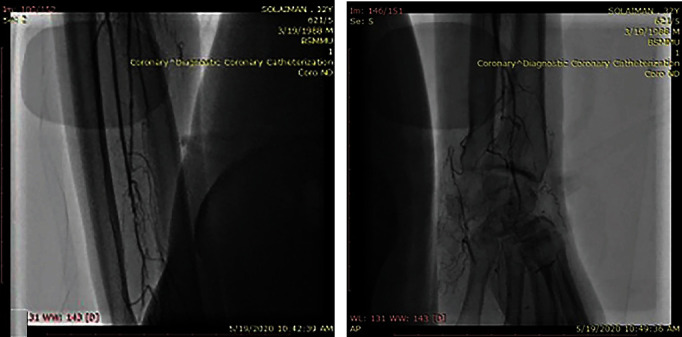
(a) Right ulnar artery nonvisualized from the midforearm and significant occlusion of the radial artery at the distal forearm; (b) distal ulnar and radial arteries are nonvisualized and very poor filling of the superficial palmar arch.

A CT scan of the abdomen confirmed the presence of hepatomegaly (18 cm); splenomegaly (12 cm); multiple lymphadenopathies involving the para-aortic region, along the coeliac axis, along both common iliac vessels, their tributaries, and the mesentery vessels; and enlarged lymph nodes in both inguinal regions (Figures 3) and 3(b)). The duplex examination of both lower limbs showed moderate to severe blood flow reduction in all distal arteries of both upper and lower limbs. No atherosclerotic plaques were seen along iliofemoral or brachial arteries. The confluent diminished blood flow was confirmed by a peripheral angiogram, which revealed a 90-95% stenosis of the right radial artery extending from the mid to distal part and also in the distal part of the left ulnar artery and the left middle posterior tibial artery. There was a 95-99% stenosis in the proximal portion of the left radial and the right ulnar arteries and a 99-100% stenosis involving the proximal part of the right posterior tibial artery and the left anterior tibial artery. There was no contrast flow after the middle part of the right anterior tibial artery and the left common peroneal artery. No skip lesions, corkscrew collaterals, or microaneurysms were observed. There was no proximal lesion (Figures 4(a), 4(b), 5(a), and 5(b)).

**Figure 6:**
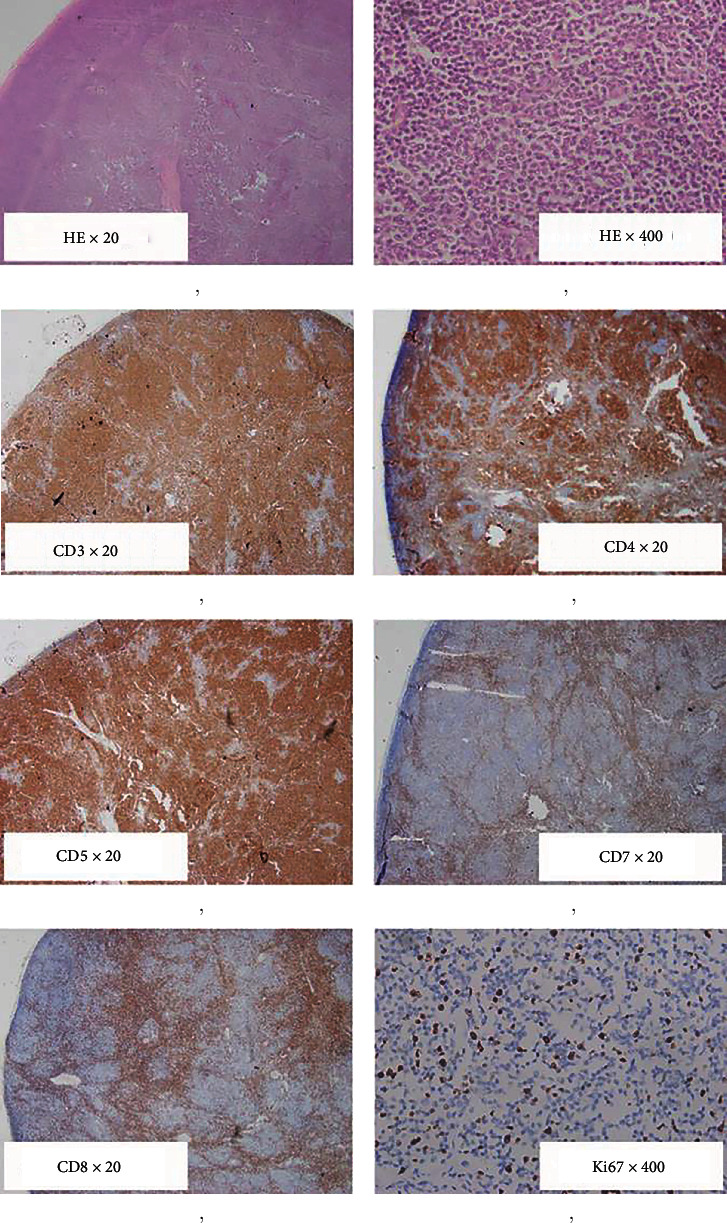
Photomicrographs of histopathology (a, b) and immunohistochemistry (c–h) of the excised cervical lymph node. CD2 staining and B cell markers are not shown. The legends are expressed as [stain] × [total magnification].

The histopathology of an excised cervical lymph node revealed that the nodal architecture was affected by diffuse proliferation of intermediate-sized atypical cells (Figure 6(b)). The conclusion of the pathologist was lymphoproliferative disorder favouring non-Hodgkin lymphoma. Immunohistochemical examination revealed positive staining for CD2, CD3, CD4, and CD5 as well as a loss of CD7 and CD8 in atypical lymphocytes along with negative staining for B cell markers, thus subtyped as peripheral T cell lymphoma, not otherwise specified (PTCL, NOS); Ki67 was positive in 35% of the atypical cells (Figures 6(c)–6(h)). Flow cytometry was not done due to unavailability.

According to the Lugano classification, the patient had stage IV disease and no systemic B symptoms.

The patient was put on three weekly cyclophosphamide, doxorubicin, vincristine, and prednisolone (CHOP 21) regimen. Initially, he was treated with low molecular weight heparin and aspirin 75 mg daily and nifedipine 20 mg twice daily without any improvement of the ischemia. An NSAID (naproxen), tramadol, and morphine (10 mg three times daily) were tried for pain relief with an inadequate response. The patient was discharged home after chemotherapy and advised for follow-up for treatment with subsequent cycles of chemotherapy. He experienced partial abatement of his symptoms for about 15 days after discharge. The second dose (CHOP 21) was interrupted for more than three months because of the prolonged lockdown due to the COVID-19 pandemic. In the meantime, gangrene of the lower limbs progressed, and the pain became excruciating. So, the patient got himself admitted into another hospital and underwent surgical amputation of gangrenous parts of all the digits and toes. The patient eventually was discharged home. We had tried to contact the patient several times by telephone, but he did not respond. Later, we were informed that he died at home in mid-July 2020, shortly after discharge from the other hospital.

## 3. Discussion and Conclusions

Lymphomas are generally divided into two broad groups of neoplasms: Hodgkin lymphoma and non-Hodgkin lymphoma. Non-Hodgkin lymphomas (NHLs) are tumours that originate from lymphoid tissues, mainly of lymph nodes. Approximately 85 percent of all malignant lymphomas are non-Hodgkin lymphomas [7]. According to the World Health Organization (WHO) classification, NHL is divided into NHL of B cell, T cell, and natural killer (NK) cell origin. There is a broad spectrum of clinical presentations according to the histologic subtype and site of involvement. Considering the prognosis and typical presentation, NHL can be divided into two groups, aggressive (e.g., diffuse large B cell lymphoma, Burkitt lymphoma, precursor B and T lymphoblastic leukaemia/lymphoma, adult T cell leukaemia-lymphoma, and certain other peripheral T cell lymphomas) and indolent (follicular lymphoma, chronic lymphocytic leukaemia/small lymphocytic lymphoma, etc.) [8]. Besides the usual nodal presentation, a small proportion of patients initially present with extranodal presentation (primary extranodal non-Hodgkin lymphoma). The systems involved in primary extranodal NHL are the GI tract (primary gastrointestinal tract lymphoma), CNS (primary central nervous system lymphoma), and integumentary system (primary cutaneous lymphoma that presents as rash, cutaneous plaques, tumour, or ulcer).

At first admission, we considered different causes for digital gangrene. The history of multiple substance abuse, including smoking and generalized lymphadenopathy, made the clinical scenario complex. Cannabis arteritis, amphetamine-induced vasculopathy, peripheral arterial disease, thromboangiitis obliterans, digital gangrene secondary to connective tissue disease, or vasculitis were the possibilities. However, histological examination led us to the final diagnosis of non-Hodgkin lymphoma. Immunohistochemical staining confirmed peripheral T cell lymphoma diagnosis, not otherwise specified (PTCL, NOS). The incidence of PTCL is higher in Asia than in the Western countries, which is 15 to 20 percent of all NHLs, and approximately 20 to 25 percent of those cases are classified as PTCL, NOS [9]. The clinical course of PTCL, NOS is aggressive with frequent relapses. The patients generally have a worse prognosis compared to those with B cell NHL. The most frequently involved extranodal sites of PTCL, NOS are the skin and gastrointestinal tract [10]. Wallett et al. described the cutaneous manifestations of PTCL, NOS in their case series, including diffuse maculopapular rash, ulcer, and urticarial plaque [11]. There are scanty reports (three) in the literature about the association of digital ischemia and non-Hodgkin lymphoma (T cell lymphoma). To our knowledge, this report describes the first patient with peripheral T cell lymphoma (not otherwise specified) in the literature, presenting with digital gangrene involving all the digits of the upper and lower limbs.

Amphetamine-like drugs have been associated with the emergence of Raynaud's phenomenon and vasculopathy by enhancing vasoconstriction [12]. The abuse of yaba, a Thai word that stands for “crazy medicine,” is rampant in Bangladesh and entire southeast Asia. The use of yaba has gained momentum among the young generation and the affluent society. It is estimated that there are about 4.6 million yaba users in Bangladesh [13]. These pills are synthetically produced and contain 25 to 35 mg of methamphetamine and 45 to 65 mg of caffeine [14]. The most prevalent amphetamine and related derivatives and analogues (ADRA) are associated with CNS and cardiac side effects [15, 16]. The peripheral vascular adverse events like Raynaud's phenomenon as a complication of ADRA abuse were previously reported in the pediatric population [17, 18]. Tan et al. published a case series comprising adults with peripheral vascular events associated with ADRA [19]. In this series, the most common presentation was mild vasospastic symptoms like acrocyanosis and Raynaud's phenomenon, but critical limb ischemia has been described in only six patients. The risk factors for the development of severe vascular manifestations like ischemia and gangrene were the presence of an underlying rheumatic disease (systemic sclerosis, systemic lupus erythematosus), female sex, or the presence of one or more cardiovascular risk factors (diabetes, hypertension, chronic kidney disease, and or tobacco use) [19]. Moreover, the severe or critical limb ischemia cases described in the abovementioned case series involved either upper or lower limbs, not both. These patients continued to use ADRA despite being diagnosed with ADRA-induced vasculopathy. For our patient, the possibility of methamphetamine/ADRA-induced digital gangrene is less likely. The patient discontinued yaba about 40 days before admission, at the initial stage of the vascular phenomenon, which progressed to the peak involving all digits leading to amputation, despite stopping the substance. He had no clinical or laboratory evidence of systemic autoimmune inflammatory diseases or any cardiovascular risk factor. The patient did not display any other clinical evidence of amphetamine toxicity like diaphoresis, hypertension, tachycardia, agitation, or psychosis. He had no preceding history of Raynaud's phenomenon. He had digital gangrene involving all the digits of both the upper and lower limbs. His urinary amphetamine molecule level was zero indicating that he quitted yaba already for a long time, as the time frame to remain positive for urine assay is 2 to 4 days for chronic exposure [20]. We cannot exclude a possible role of yaba in our patient; however, the association with NHL appears the most likely explanation when considering these data, particularly in the presence of lymphadenopathy and organomegaly.

Cannabis (also called marijuana) may involve the peripheral vascular system, which is termed cannabis arteritis. This condition may present with Raynaud's phenomenon, ulceration, or necrosis in the digits or distal extremities [21]. The first case of arteritis in a young cannabis abuser was reported in 1960 [22]. Various symptoms described in the literature have been summarised in a review like claudication, acral pain, Raynaud's phenomenon, subacute digital ischemia of the lower limbs, distal necrosis or gangrene of the lower limbs, early disappearance of the distal pulses, and sometimes venous thrombosis (Peyrot et al. [23]). The most effective treatment of cannabis arteritis is the complete withdrawal of cannabis consumption, halting the progression [23, 24]. As previously mentioned, our patient had no history of Raynaud's phenomenon, and the digital gangrene involved both upper and lower limbs. Cannabis can be detected for a long time in chronic abusers (≥1 month) in the urine drug testing assays to detect drugs of abuse [20]. A negative screening indicated he quit smoking marijuana for at least a month before admission. The digits' distal necrosis progressed even though our patient had discontinued using cannabis when he noticed the first symptoms. So, we cannot exclude the role of cannabis in our patient's disease, but the fact that symptoms did not improve after quitting cannabis does not support this. Moreover, the presence of generalized lymphadenopathy and hepatosplenomegaly favours NHL.

Buerger's disease (thromboangiitis obliterans or TAO) is a nonatherosclerotic segmental inflammatory thrombosis of the distal upper and lower limb arteries, which may occur in young male tobacco smokers [25]. The prevalence of smoking in Bangladesh varies from 39 to 73% among adult males in different urban and semiurban settings [26]. TAO generally occurs in heavy cigarette smokers who usually smoked an average of 23 years [27]. Buerger's disease is common in Bangladesh due to the widespread availability of different types of smoking materials like cigarettes and bidi (a cheaper substitute for a cigarette containing a higher amount of nicotine than a cigarette) [26]. Superficial migratory thrombophlebitis may predate the onset of TAO's typical features like ischemic pain in the digits and distal acral gangrene [28]. Secondary Raynaud phenomenon can occur in more than 40% of patients with TAO, whereas arthralgia and arthritis (mostly in knees and wrists) may precede the vascular occlusive features in 12.5% of the patients [29]. The typical angiographic findings of TAO-like segmental occlusion of diseased segment interlaced between normal-appearing segments with the development of collateral vessels around the areas of occlusion (i.e., corkscrew collaterals) [29] were absent in this patient. Complete cessation of smoking is the only way to halt the progression of thromboangiitis obliterans, and the requirement for amputation becomes quite low [30]. Amputation can be avoided in 94% of those who quit smoking [31]. These antecedents mentioned above were absent in our patient. He did not have the arteriographic findings suggestive of thromboangiitis obliterans. The digital gangrene advanced despite the termination of smoking more than a month before admission at the initial stage of digital infarction. Ultimately, amputation of all the digits was necessary. Thus, we excluded the diagnosis of TAO in our patient.

The peripheral arterial disease (PAD) can be another possibility for a male presenting with digital ischemia. PAD mainly occurs after 40 years on the background of some traditional risk factors like diabetes, hypertension, dyslipidemia, smoking, family history of atherosclerosis, or premature cardiovascular events. Apart from smoking, this patient did not have any of these risk factors. There was no history of intermittent claudication. The vascular survey revealed the absence of a bruit and no significant blood pressure difference between the arms, and the Berger test was negative. No proximal lesion was detected in the arteriography or significant atherosclerotic plaque in the duplex study of the vessels.

Some of the important differential diagnoses of digital ischemia are secondary Raynaud's phenomenon due to connective tissue diseases or autoimmune inflammatory diseases, vasculitis, antiphospholipid syndrome, etc. Raynaud's phenomenon was absent in this patient. There were no clinical indications of systemic inflammatory conditions like a rash, fever, myalgia, arthralgia or arthritis, weakness, dryness of mouth or eyes, skin tightening, cough, and shortness of breath. There was no previous history suggestive of arterial or venous thrombosis. The acute phase reactants like erythrocyte sedimentation rate and C-reactive protein were normal. The chest X-ray was normal. The serology for hepatitis B and C was negative, and autoantibodies were negative, suggesting a rheumatic condition. The angiographic features of polyarteritis nodosa, like stenosis and/or microaneurysm, were not demonstrated in the peripheral angiography. So, there were no indications that our patient had an autoimmune vascular disorder.

The exclusion of other possibilities of digital gangrene through clinical and laboratory evaluation and the presence of lymphadenopathy led us to consider a paraneoplastic aetiology. The presence of acral vascular syndromes is observed in several malignant conditions, mostly in solid tumours as adenocarcinomas (lung, gastrointestinal tract, and breast) followed by haematooncological diseases, predominantly lymphoproliferative disorders [4]. The acral vascular syndromes can cooccur, predate, or present after the diagnosis of malignancy [32]. Although an uncommon finding with sparse data in the literature, digital ischemia may occur as a paraneoplastic feature of T cell non-Hodgkin lymphoma [33, 34]. The pathophysiology of paraneoplastic vascular syndrome is poorly understood and likely to be multifactorial. Paraneoplastic acral vascular syndromes equally affect both sexes, whereas primary Raynaud's phenomenon or secondary Raynaud's due to connective tissue diseases occurs predominantly in the young female population. Vasoconstrictor substances released by the tumour cells may cause ischemia [35]. Acquired thrombogenic coagulation abnormalities are common in different malignancies, such as prolonged partial thromboplastin time, thrombocytosis, or elevated cryofibrinogen levels [36–38]. A vasculitic process secondary to immune complex deposition may be another responsible factor [35]. Compression of the cervical plexus by the tumour or the metastasized lymph node can hyperstimulate the sympathetic nervous system causing acral vasoconstriction [39]. Treatment of the underlying malignancy may improve the vascular symptoms in most cases [4].

Our case illustrates a case of non-Hodgkin lymphoma presenting with digital gangrene, which ultimately required amputation. Multiple confounding factors like smoking, cannabis, and methamphetamine abuse made the clinical scenario complicated. Progressive worsening of digital ischemia despite the cessation of smoking, marijuana, and methamphetamine abuse leading to amputation pointed to the diagnosis of some endogenous cause. A case report recently published on Lennert-type T cell lymphoma presenting as digital gangrene in the right middle finger with some necrotic areas over the shin [40]. This article may be the first case report of digital gangrene in a patient with non-Hodgkin lymphoma (peripheral T cell lymphoma, not otherwise specified) with concomitant cannabis and methamphetamine abuse involving all the digits. It may hint at a possibility that substance abuse may predispose a patient with T cell NHL to this paraneoplastic complication.

A limitation of this case report was that we could not observe the complete response to treatment of the underlying NHL, as the treatment was interrupted due to the prolonged lockdown for COVID-19, and the patient expired.

In conclusion, malignancy-associated acral vascular syndrome is an infrequent phenomenon that may mimic vasculitis is an infrequent phenomenon. A paraneoplastic occurrence should be considered on the background of rapid onset digital ischemia in a male patient without atherogenic or thrombotic risk factors, Raynaud's phenomenon or autoimmune disorders, or vasoconstrictive drugs. A high index of suspicion is necessary for proper diagnosis as delay in diagnosis will yield poorer treatment outcomes.

## Data Availability

We prefer to deposit our data in a public repository that meets appropriate standards of archiving, citation, and curation. The findings of this paper should be publicly available whenever possible and as open as possible.
